# A species of the genus *Panophrys* (Anura, Megophryidae) from southeastern Guizhou Province, China

**DOI:** 10.3897/zookeys.1047.61097

**Published:** 2021-06-24

**Authors:** Tao Luo, Yali Wang, Siwei Wang, Xueli Lu, Weifeng Wang, Huaiqing Deng, Jiang Zhou

**Affiliations:** 1 School of Karst Sciences, Guizhou Normal University, Guiyang 550001, Guizhou, China Guizhou Normal University Guiyang China; 2 School of Life Sciences, Guizhou Normal University, Guiyang 550001, Guizhou, China Guizhou Normal University Guiyang China

**Keywords:** Morphology, new species, *
Panophrys
*, southwest China, taxonomy

## Abstract

Herein, we describe *Panophrys congjiangensis***sp. nov.** obtained from the Yueliangshan Nature Reserve, Congjiang County, Guizhou Province, China. Phylogenetic analyses based on the mitochondrial genes 16S rRNA and COI indicated that this new species represented an independent lineage, closely related to *P. leishanensis*. The uncorrected genetic distances between the new species and its closest congener, *P. leishanensis*, were 3.0% for 16S rRNA and 8.4% for COI. The new species is distinguished from its congeners by a combination of the following morphological characteristics (1) medium body size (SVL 28.6–33.4 mm in males and 38.4–40.2 mm in females); (2) a small horn-like tubercle at the edge of each upper eyelid; (3) the tympanum distinctly visible (TD/ED ratio 0.47–0.66); (4) vomerine teeth absent; (5) the tongue not notched behind; (6) a narrow and unobvious lateral fringe on toes; (7) relative finger lengths II < I < V < III; (8) rudimentary webs on toes; (9) hindlimbs slender, heels overlapping when thighs are positioned at right angles to the body; (10) two metacarpal tubercles on the palm, with the inner metatarsal tubercle long and oval-shaped; (11) the tibiotarsal articulation reaching the nostril when the leg is adpressed and stretched forward; (12) dorsal skin rough with numerous orange–red granules, ventral surface smooth; (13) a single internal subgular vocal sac present in males; and (14) in breeding males, weak gray-black nuptial pads with black nuptial spines present on the dorsal surface of the bases of the first and second fingers. To date, the new species is only known from the type locality.

## Introduction

The Asian horned toad subfamily Megophryinae (Bonaparte, 1850) is widely distributed in southern China, the southern and eastern Himalayas, and across Indochina to the islands of the Sunda Shelf and the Philippines (Fei and Ye 2016; Mahony et al. 2017; Liu et al. 2018; Frost 2021). The widespread distributions and morphological similarities of subfamily Megophryinae species have long made the discrimination of species in this subfamily controversial (Dubois 1987 “1986”; Rao and Yang 1997; Dubois and Ohler 1998; Jiang et al. 2003; Zheng et al. 2004; Frost et al. 2006; Li and Wang 2008; Fei et al. 2009; Fei and Ye 2016; Chen et al. 2017; Mahony et al. 2017). In recent phylogenetic analyses, subfamily Megophryinae has been recognized as a broadly monophyletic genus (i.e., *Megophrys* sensu lato; Chen et al. 2017; Mahony et al. 2017; Liu et al. 2018). These revisions have recommended that subfamily Megophryinae be divided into seven subgenera under the genus *Megophrys* sensu lato: *Atympanophrys* Tian & Hu, 1983; *Brachytarsophrys* Tian & Hu, 1983; *Megophryss.s* Kuhl & Van Hasselt, 1822; *Ophryophryne* Boulenger, 1903; *Pelobatrachus* Beddard, 1908; *Panophrys* Rao & Yang, 1997; and *Xenophrys* Günther, 1864. To resolve these classification conflicts, Li et al. (2020a) suggested to elevate the seven monophyletic subgenera by Mahony et al. (2017) to the level of genera, namely: *Atympanophrys*, *Brachytarsophrys*, *Megophrys*, *Ophryophryne*, *Panophrys*, *Pelobatrachus*, and *Xenophrys*. In this study, we have followed this recommendation (Li et al. 2020a; Frost 2021).

In the most recent revision, 59 species were assigned to the genus *Panophrys* (Frost 2021), 40 of these species were described in the past decade. In total, 55 *Panophrys* species have been described from China (see *Panophrys* species list in Frost 2021), and 11 are known specifically from Guizhou Province, i.e., *Panophrys anlongensis* (Li, Lu, Liu & Wang, 2020), *Panophrys binlingensis* (Ye & Fei, 1995), *Panophrys chishuiensis* (Xu, Li, Liu, Wei & Wang, 2020), *Panophrys jiangi* (Liu, Li, Wei, Xu, Cheng, Wang & Wu, 2020), *Panophrys leishanensis* (Li, Xu, Liu, Jiang, Wei & Wang, 2018), *Panophrys liboensis* (Zhang, Li, Xiao, Li, Pan, Wang, Zhang & Zhou, 2017), *Panophrys omeimontis* (Liu, 1950), *Panophrys shuichengensis* (Tian & Sun, 1995), *Panophrys spinata* (Liu & Hu, 1973), *Panophrys platyparietus* (Rao & Yang, 1997), and *Panophrys qianbeiensis* (Su, Shi, Wu, Li, Yao, Wang & Li, 2020). All of these species inhabit isolated mountain streams in evergreen broadleaf forests in Guizhou Province. Such isolated conditions may be favorable for species formation. For example, *P. spinata* has historically been recorded from several counties in Guizhou Province (Dafang, Jinsha, Suiyang, Jiangkou, Yinjiang, and Leishan; Wu et al. 1986; Fei et al. 2009). A recent phylogenetic analysis showed that the Suiyang population, originally recorded as *P. spinata*, was genetically closer to *P. spinata* and *Panophrys sangzhiensis* (Jiang, Ye & Fei, 2008). Thus, the Suiyang population was described as a new species, *P. qianbeiensis* (Su et al. 2020). Therefore, the diversity of *Panophrys* may be greater in Guizhou Province than is currently assumed.

**Figure 1.**
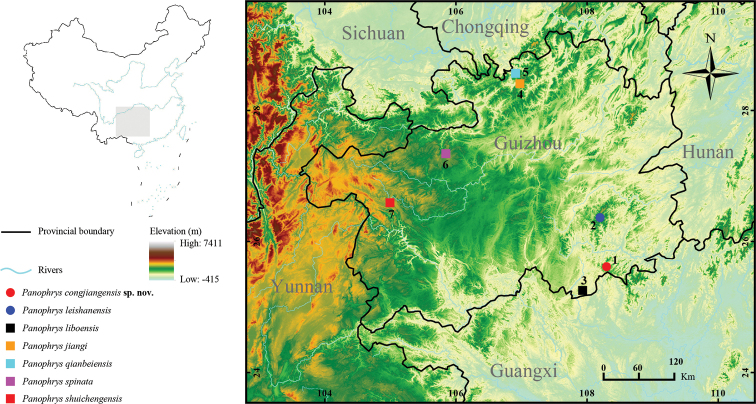
Sampling collection localities and distribution of the *Panophrys congjiangensis* sp. nov., *P. leishanensis*, *P. liboensis*, and *P. jiangi* in Guizhou Province, China **1** Yueliangshan Nature Reserve, Congjiang County, Guizhou Province **2** Leigongshan National Nature Reserve, Leishan County, Guizhou Province **3** Maolan National Nature Reserve, Libo County, Guizhou Province **4, 5** Huoqiuba Nature Reserve, Suiyang County, Guizhou Province **6** Xingxiu Township, Dafang County, Guizhou Province **7** Fenghuang Township, Shuicheng County, Guizhou Province. The base maps are from Standard Map Service website (http://bzdt.ch.mnr.gov.cn/index.html).

During herpetological surveys conducted between 2019 and 2020 in Yueliangshan Nature Reserve, Congjiang County, Guizhou Province, China (Fig. 1), we captured several specimens of an unknown anuran species. Based on morphological characteristics, including body size (i.e., body length < 45 mm) and a small horn-like tubercle at the middle edge of each upper eyelid, these specimens were identified as a species of *Panophrys*, initially *P. minor* (Fei et al. 2009; Fei and Ye 2016). However, subsequent observation indicated that these newly collected specimens differed from any currently described *Panophrys* species. Indeed, molecular phylogenetics, comparative morphology, and bioacoustics data suggest that these specimens represent a previously unknown species. This new species is described herein.

## Material and methods

### Sampling

A total of 25 specimens were collected in this study: 22 were collected in Congjiang County, Guizhou Province, China, and were identified as an unknown species. The remaining 3 specimens, collected in Kuankuoshui National Nature Reserve, Suiyang County, Guizhou Province, China, were identified as *P. jiangi*. All specimens were fixed in 10% buffered formalin and later transferred to 75% ethanol for preservation. The muscle samples used for molecular analysis were preserved in 95% alcohol and stored at -20 °C. All specimens are housed at Guizhou Normal University (**GZNU**), Guiyang City, Guizhou Province, China.

### DNA extraction, PCR, and sequencing

Genomic DNA was extracted from the muscle tissue samples using DNA extraction kits (Tiangen Biotech (Beijing) Co., Ltd.). We amplified and sequenced two mitochondrial genes from each DNA sample: partial 16S ribosomal RNA (16S rRNA), using primers L3975 (5’-CGCCTGTTTACCAAAAACAT-3’) and H4551 (5’-CCGGTCTGAACTCAGATCACGT-3’) following Simon et al. (1994); and cytochrome C oxidase I (COI), using primers Chmf4 (5’-TYTCWACWAAYCAYAAAGAYATCGG-3’) and Chmr4 (5’-ACYTCRGGRTGRCCRAARAATAATCA-3’) following Che et al. (2012). PCR ampliﬁcations were performed in 25 μL reaction volumes with the following cycling conditions: an initial denaturing step at 95 °C for five min; 36 cycles of denaturation at 95 °C for 40 s, annealing at 52 °C (for 16S rRNA) or 47 °C (for COI) for 40 s, then extension at 72 °C for 1 min; and a ﬁnal extension step at 72 °C for 10 min. The purified PCR products were sequenced with both forward and reverse primers using BigDye Terminator Cycle Sequencing Kits, following the manufacturer’s instructions, on an ABI Prism 3730 automated DNA sequencer by Tsing KE Biological Technology Co. Ltd. (Chengdu, China). All sequences have been deposited in GenBank (Table 1).

**Table 1.** Localities, voucher information, and GenBank numbers for all samples used in this study.

ID	Species	Locality	Voucher number	Genbank accession No.	
				16S rRNA	COI
1	*Panophrys obesa*	Heishiding Nature Reserve, Guangdong, China	SYS a002271	KJ579121	MH406123
2	*Panophrys ombrophila*	Wuyi Shan, Fujian, China	WUYI2015101	KX856397	–
3	*Panophrys cheni*	Taoyuandong Nature Reserve, Hunan, China	SYS a002123	KJ560396	MF667904
4	*Panophrys dongguanensis*	Yinping Shan, Guangdong, China	SYS a002007	MH406654	MH406090
5	*Panophrys nankunensis*	Nankun Shan, Guangdong, China	SYS a004498	MK524108	MK524139
6	*Panophrys wugongensis*	Wugongshan Scenic Area, Jiangxi, China	SYS a002610	MK524114	MK524145
7	*Panophrys insularis*	Nan’ao Island, Guangdong, China	SYS a002169	MF667887	MF667924
8	*Panophrys lini*	Nanfengmian Nature Reserve, Jiangxi, China	SYS a002128	KJ560416	MF667907
9	*Panophrys nanlingensis*	Nanling Nature Reserve, Guangdong, China	SYS a001959	MK524111	MK524142
10	*Panophrys xiangnanensis*	Yangming Shan, Hunan, China	SYS a002875	MH406714	MH406166
11	*Panophrys baishanzuensis*	Baishanzu National Park, Qingyuan, Zhejiang, China	CIBQY20200719001	MW001150	MT998291
12	*Panophrys brachykolos*	Hongkong, China	SYS a005563	MK524122	MK524153
13	*Panophrys kuatunensis*	Wuyi Shan, Jiangxi, China	SYS a003449	MF667881	MF667916
14	*Panophrys lishuiensis*	Lishui City, Zhejiang, China	CIBWYF00169	KY021418	–
15	*Panophrys xianjuensis*	Xianju, County, Zhejiang, China	CIBXJ20190801	MN563754	MN563770
16	*Panophrys jinggangensis*	Jinggang Shan, Jiangxi, China	SYS a004028	MH406780	MH406239
17	*Panophrys liboensis*	Libo Country, Guizhou, China	GZNU20150813001	MF285253	MW959767
18		Libo Country, Guizhou, China	GZNU20160408007	MF285258	MW959768
19		Libo Country, Guizhou, China	GZNU20160408006	MF285257	MW959769
20		Libo Country, Guizhou, China	GZNU20160408004	MF285256	MW959770
21	*Panophrys boettgeri*	Longhu Forest Station, Fujian, China	SYS a004126	MH406785	MH406245
22	*Panophrys huangshanensis*	Huang Shan, Anhui, China	SYS a002702	MF667882	MF667919
23	*Panophrys congjiangensis* sp. nov.	Yueliangshan Nature Reserve, Congjiang, Guizhou, China	GZNU20200706003	MW959773	MW959761
24		Yueliangshan Nature Reserve, Congjiang, Guizhou, China	GZNU20200706004	MW959774	MW959762
25		Yueliangshan Nature Reserve, Congjiang, Guizhou, China	GZNU20200706005	MW959775	MW959763
26		Yueliangshan Nature Reserve, Congjiang, Guizhou, China	GZNU20200706000	MW959776	MW959764
27	*Panophrys leishanensis*	Leigong Shan, Guizhou, China	CIBLS20141004003	MK005308	MK005304
28		Leigong Shan, Guizhou, China	SYSa002213	MH406673	MH406113
29		Leigong Shan, Guizhou, China	CIBLS20160610005	MK005309	MK005305
30	*Panophrys baolongensis*	Baolong, Chongqing, China	KIZ019216	KX811813	KX812093
31	*Panophrys wushanensis*	Shennongjia Nature Reserve, Hubei, China	SYS a003008	MH406732	MH406184
32	*Panophrys tuberogranulata*	Badagong Shan, Hunan, China	SYS a004310	MH406801	MH406263
33	*Panophrys shimentaina*	Shimentai Nature Reserve, Guangdong, China	SYS a002078	MH406656	MH406093
34	*Panophrys yangmingensis*	Yangming Shan, Hunan, China	SYS a002889	MH406720	MH406172
35	*Panophrys jiulianensis*	Nankun Shan, Guangdong, China	SYS a003623	MK524103	MK524134
36	*Panophrys mirabilis*	Huaping Nature Reserve, Guangxi, China	SYS a002193	MH406670	MH406110
37	*Panophrys shunhuangensis*	Nanshan National Forest Park, Hunan, China	HNNU18NS01	MK836023	MK977594
38	*Panophrys acuta*	Heishiding Nature Reserve, Guangdong, China	SYS a002159	MF667869	MF667899
39	*Panophrys mufumontana*	Mufu Shan, Hunan, China	SYS a006390/CIB110012	MK524104	MK524135
40	*Panophrys caudoprocta*	Badagong Shan, Hunan, China	SYS a004281	MH406795	MH406257
41	*Panophrys sangzhiensis*	Badagong Shan, Hunan, China	SYS a004307	MH406798	MH406260
42	*Panophrys spinata*	Leigong Shan, Guizhou, China	SYS a002226	MH406675	MH406115
43	*Panophrys qianbeiensis*	Huanglian Nature Reserve, Guizhou, China	CIBTZ20190608015	MT651553	MT654520
44	*Panophrys binlingensis*	Wawu Shan, Sichuan, China	SYS a005313	MH406892	MH406354
45	*Panophrys binchuanensis*	Jizu Shan, Yunnan, China	KIZ019441	KX811849	KX812112
46	*Panophrys angka*	Kiew Mae Pan nature trail, Chiang Mai, Thailand	KIZ040591	MN508052	–
47	*Panophrys anlongensis*	Anlong County, Guizhou, China	CIBAL20190531018	MT823184	MT823261
48	*Panophrys omeimontis*	Laojun Shan, Sichuan, China	SYS a002741	MH406710	MH406162
49	*Panophrys palpebralespinosa*	Pu Hu Nature Reserve, Thanh Hoa, Vietnam	KIZ011603	KX811888	KX812137
50	*Panophrys caobangensis*	Nguyen Binh, Cao Bang,Vietnam	IEBR 4385	LC483945	–
51	*Panophrys daweimontis*	Dawei Shan, Yunnan, China	KIZ048997	KX811867	KX812125
52	*Panophrys jingdongensis*	Wuliang Shan, Yunnan, China	SYS a003928	MH406773	MH406232
53	*Panophrys rubrimera*	Lao Cai, Sa Pa, Vietnam	AMSR177676	MF536419	–
54	*Panophrys wuliangshanensis*	Wuliang Shan, Yunnan, China	SYS a003924	MH406771	MH406230
55	*Panophrys fansipanensis*	Lao Cai, Sa Pa, Vietnam	VNMN 2018.01	MH514886	–
56	*Panophrys hoanglienensis*	Lao Cai, Sa Pa, Vietnam	VNMN 2018.02	MH514889	–
57	*Panophrys jiangi*	Huoqiuba Nature Reserve, Guizhou, China	GZNU20180606020	MW959777	MW959765
58		Kuankuosui Nature Reserve, Guizhou, China	GZNU20070712001	MW959778	MW959766
59		Kuankuosui Nature Reserve, Guizhou, China	CIBKKS20180722006	MN107743	MN107748
60	*Panophrys minor*	Qingcheng Shan, Sichuan, China	SYS a003209	MF667862	MF667891
61	*Panophrys chishuiensis*	Chishui, County, Guizhou, China	CIBCS20190518031	MN954707	MN928958
62	*Ophryophryne pachyproctus*	Beibeng, Xizang, China	KIZ010978	KX811908	KX812153
63	*Panophrys yeae*	Didong, Medog, Tibet, China	CIB201706MT01	MN963217	MN964312
64	*Panophrys zhoui*	Renqinbeng, Medog, Tibet, China	CIBMT171053	MN963207	MN964322
65	*Xenophrys vegrandis*	West Kameng district, Arunachal Pradesh, India	ZSI A 11605	KY022305	MH647530
66	*Ophryophryne elfina*	Bidoup Mountain, Lam Dong, Vietnam	ZMMU ABV000454	KY425379	–
67	*Ophryophryne gerti*	Nui Chua National Park, Ninh Thuan, Vietnam	ITBCZ 1108	KX811917	KX812161
68	*Ophryophryne synoria*	O’Reang, Mondolkiri, Cambodia	FMNH 262779	MN629394	–
69	*Ophryophryne hansi*	Phong Dien Nature Reserve, Thua Thien Hue, Vietnam	KIZ010360	KX811913	KX812155
70	*Ophryophryne microstoma*	Wuhuang Shan,, Guangxi, China	SYS a003492	MK524125	MK524156
71	*Megophrys montana*	Sukabumi, Java, Indonesia	LSUMZ 81916	KX811927	KX812163
72	*Megophrys parallela*	–	RMAS 021	KY679897	–
73	*Megophrys lancip*	Ulubelu, Ngarip, Indonesia	MZB:Amp:22233	KY679891	–
74	*Xenophrys medogensis*	Medog County, Tibet, China	SYS a002932	MH406725	MH406177
75	*Xenophrys robusta*	Darjeeling dist, West Bengal, India	SDBDU 2011.1057	KY022314	MH647535
76	*Xenophrys glandulosa*	Gaoligong Shan,, Yunnan, China	SYS a003758	MH406755	MH406214
77	*Xenophrys himalayana*	East Siang dist, Arunachal Pradesh, India	SDBDU2009.75	KY022311	
78	*Xenophrys periosa*	East Siang dist, Arunachal Pradesh, India	BNHS 6061	KY022309	MH647528
79	*Xenophrys monticola*	Darjeeling dist, West Bengal, India	SDBDU 2011.1047	KX894679	
80	*Xenophrys zhangi*	Zhangmu, Xizang, China	KIZ014278	KX811765	KX812084
81	*Xenophrys flavipunctata*	Hills dist, East Khasi, Meghalaya	SDBDU2009.297	KY022307	MH647536
82	*Xenophrys mangshanensis*	Longtou *glandulosa*, Guangdong, China	SYS a002750	MF667866	MF667895
83	*Xenophrys maosonensis*	Xiaoqiaogou Nature Reserve, Yunnan, China	KIZ016045	KX811780	KX812080
84	*Xenophrys oreocrypta*	West Garo Hills dist, Meghalaya	BNHS 6046	KY022306	–
85	*Xenophrys major*	Zhushihe, Yunnan, China	SYSa002961	MH406728	MH406180
86	*Xenophrys awuh*	–	SDBDU2007.161	KY022319	–
87	*Xenophrys serchhipii*	North dist, Tripura, India	SDBDU 2009.612	KY022323	MH647532
88	*Xenophrys zunhebotoensis*	–	SDBDU 2009.110	KY022321	
89	*Xenophrys ancrae*	Changlang dist, Arunachal Pradesh, India	SDBDU 2009.727	KY022318	MH647531
90	*Xenophrys numhbumaeng*	–	SDBDU 2007.041	KY022316	
91	*Xenophrys oropedion*	Mawphlang Sacred Forest, Meghalaya, India	SDBDU 2009.299	KY022317	MH647534
92	*Xenophrys megacephala*	–	ZSI A 11213	KY022315	MH647533
93	*Xenophrys dzukou*	–	SDBDU2007.106	KY022324	–
94	*Xenophrys lekaguli*	Pang Si Da National Park, Sa Kaeo, Thailand	FMNH 265955	KY022214	–
95	*Xenophrys takensis*	–	FMNH 261711	KY022215	–
96	*Xenophrys auralensis*	Aural, Kampong Speu, Cambodia	NCSM 79599	KX811807	–
97	*Xenophrys parva*	Zhushihe, Yunnan, China	SYSa003042	MH406737	MH406189
98	*Xenophrys aceras*	Khao Nan National Park, Nakhon Si Thammarat, Thailand	KIZ025467	KX811925	KX812159
99	* Xenophryslongipes *	Genting highland, Malaysia	IABHU 21101	AB530656	–
100	*Atympanophrys gigantica*	Ailao Shan, Yunnan, China	SYS a003883	MH406766	MH406225
101	*Atympanophrys shapingensis*	Wawu Shan, Sichuan, China	SYS a005310	MH406890	MH406352
102	*Atympanophrys nankiangensis*	Nanjiang, Sichuan, China	CIB ZYC517	KX811900	–
103	*Atympanophrys wawuensis*	Wawu Shan, Sichuan, China	KIZ025799	KX811902	KX812062
104	*Brachytarsophrys feae*	Huangcaoling, Yunnan, China	KIZ046706	KX811810	KX812056
105	*Brachytarsophrys platyparietus*	–	W01395	AY526206	–
106	*Brachytarsophrys chuannanensis*	Hejiang County, Sichuan, China	SYS a004926	MH406901	MH406364
107	*Brachytarsophrys carinense*	Dayao Shan, Guangxi, China	Tissue ID: YPX20455	KX811811	KX812057
108	*Brachytarsophrys popei*	Jinggang Shan, Jiangxi, China	SYS a004209	MK524124	MK524155
109	*Brachytarsophrys intermedia*	Phong Nha0Ke Bang NP, U Bo, Vietnam	ZFMK 87596	HQ588950	–
110	“*Megophrys*”*dringi*	Mulu National Park, Sarawak Gunung, Malaysia	UNIMAS 8943	KJ831317	–
111	*Pelobatrachus baluensis*	Gunung Kinabalu National Park, Kogopan Trail, Malaysia	ZMH A13125	KJ831310	–
112	*Pelobatrachus stejnegeri*	Pasonanca Natural Park, Zamboanga, Philippines	KU 314303	KX811922	KX812052
113	*Pelobatrachus kobayashii*	Gunung, Sabah, Meghalaya	UNIMAS 8148	KJ831313	–
114	*Pelobatrachus ligayae*	Palawan, Philippines	ZMMU NAP005015	KX811919	KX812051
115	*Pelobatrachus kalimantanensis*	Kalimantan Selatan, Borneo, Indonesia	MZB. Amph 21482	MG993554	–
116	*Pelobatrachus nasuta*	Sabah, Lahad Datu District, Malaysia	FMNH 231281	KY022186	–
117	*Pelobatrachus edwardinae*	Bintulu, Sarawak, Malaysia	FMNH 273694	KX811918	KX812050
118	*Leptobrachium boringii*	Emei Shan, Sichuan, China	Tissue ID: YPX37539	KX811930	KX812164
119	*Leptobrachella oshanensis*	Emei Shan, Sichuan, China	KIZ025778	KX811928	KX812166

### Phylogenetic analyses

We used a total of 194 gene sequences (112 16S rRNA sequences and 82 COI sequences) for the molecular analyses, representing 102 species of subfamily Megophryinae. Two mitochondrial genes were sequenced in 10 muscle tissue samples from the specimens collected in this study, and 178 sequences were downloaded from GenBank. Samples included those from the undescribed species collected and named in this study (Fig. 1). Following Mahony et al. (2017), we selected *Leptobrachium boringii* (Liu, 1945) and *Leptobrachella oshanensis* (Liu, 1950) as outgroups. The two outgroup sequences were obtained from GenBank. Details of the sequences used for phylogenetic analysis are given in Table 1.

All sequences were assembled and aligned using the MUSCLE (Edgar 2004) module in MEGA 7.0 (Kumar et al. 2016) with default settings. Alignments were checked by eye and revised manually if necessary. Trimming, with gaps partially deleted, was performed using GBLOCKS 0.91b (Castresana 2000). The best-fit partitioning schemes and corresponding substitution models for the concatenated-sequence supermatrix were selected in PartitionFinder 2.1.1 using the Bayesian information criterion (Lanfear et al. 2016). As a result, the analysis suggested that the best partition scheme 16S rRNA gene/each codon position of COI gene, and selected GTR+I+G model as the best model for 16S rRNA gene, and TRNEF+G, HKY+I+G, and TIM+G model as the best model for first, second and third codons position of COI gene, respectively. Phylogenetic analysis of the concatenated-sequence matrix was performed using maximum likelihood (ML) and Bayesian inference (BI). ML and BI phylogenies based on the concatenated-sequence matrix were constructed using both IQ-tree 2.0.4 (Nguyen et al. 2015) and MrBayes 3.2.1 (Ronquist et al. 2012), according to the best-fit partitioning schemes and the corresponding substitution models. The ML analysis was performed using the best-fit model for each partition with 2000 ultrafast bootstrap (UFB) replicates (Minh et al. 2013); the analysis was continued until a correlation coefficient of at least 0.99 was reached (Hoang et al. 2018). We performed two independent BI runs using four Markov chains (three heated chains and a single cold chain). The best-fit partitioning schemes and corresponding substitution models were selected. The BI analysis started with a random tree; each run consisted of 2 × 10^7^ generations, sampled every 1000 generations. Runs were considered to have converged when the average standard deviation of split frequencies (ASDSF) was less than 0.01, and the effective sample sizes (ESS) in Tracer 1.7.1 (Rambaut et al. 2018) was greater than 200. Nodes in the trees were considered well-supported when Bayesian posterior probabilities (BPP) were ≥ 0.95 and ML ultrafast bootstrap values (UFB) were ≥ 95%. The phylogenetic trees were visualized using FigTree 1.4.3 (Rambaut 2016). The uncorrected *p*-distance model in MEGA 7.0 (Kumar et al. 2016) was used to calculate average genetic distances among species based on 16S rRNA and COI.

### Species delimitation

To assess whether new species represent a valid species, two different methods were executed. We chose to include new species in the phylogenetic tree as well as several closely related species for species delimitation analysis. First, a Bayesian hypothesis-testing approach (Bayes Factor Delimitation, BFD) was implemented to statistically test alternate hypotheses of species delimitation (Gummer et al. 2014). Two species models were tested: 11 species (contains new species) and 10 species (lump new species with *P. leishanensis*). All analyses were performed in *BEAST using BEAST 1.8.2 (Drummond et al. 2012) under an uncorrelated lognormal relaxed molecular clock. A Yule process was used for the species tree prior, and the piecewise linear and constant root was used for the population size model. Two independent runs for each model were performed in BEAST 1.8.2 to assess convergence of the MCMC runs. *BEAST was run each time for 1×10^7^ generations of the MCMC algorithm sampling every 1000 generations and discarding the first 25% of the iterations as “burn-in”. After *BEAST analyses, two methods of marginal-likelihood estimation, including path-sampling (PS; Baele et al. 2012) and stepping-stone analysis (SS; Xie et al. 2011), were performed. PS and SS analyses were each run for a chain length of 1×10^6^ generations for 100 path steps. We followed the suggestions provided by Gummer et al. (2014) to assess the strength of support for a particular species delimitation hypothesis.

In addition to the Bayesian methods tested, we also applied three tree-based species-delimitation methods, i.e., Bayesian implementation of the Poisson Tree Processes model (bPTP; Zhang et al. 2013). The parameters of these three analyses were set as follows: 1×10^5^ generations, a thinning of 100 and burn-in of 10%. Convergence of models were assessed by visualizing plots of the MCMC iteration vs. the Log likelihood results. The bPTP analysis was conducted on the bPTP web server (http://species.h-its.org/ptp/) using mtDNA-based BI gene tree as input.

### Morphological comparisons

**Table 2.** Measurements of the adult specimens of *Panophrys congjiangensis* sp. nov. All units in mm. See abbreviations for the morphological characters in the Materials and Methods section. (M = male, F = female, other abbreviations defined in text), * for the holotype.

Voucher number	Sex	SVL	HDL	HDW	SNT	ED	IOD	IND	TD	UEW	NED	TED	HND	LAHL	LW	FIL	FIIL	FIIIL	FIVL	TL	THL	FL	TFL	HLL	TW	IPTL	OPTL	IMTL
GZNU20200706010^*^	M	33.4	11.3	11.1	4.2	3.8	3.1	3.7	2.4	2.9	2.3	2.0	8.6	14.7	2.3	4.5	4.1	6.3	5.5	17.5	17.8	15.7	24.9	60.2	3.5	1.8	1.6	2.2
GZNU20200706001	M	33.1	11.2	11.4	4.2	3.5	3.2	3.7	2.3	2.8	2.5	1.9	8.3	14.3	2.1	4.7	4.2	6.1	5.2	16.7	16.5	15.6	23.6	56.8	3.2	2.1	1.5	2.3
GZNU20200706002	M	30.1	11.2	10.8	3.9	3.5	3.5	3.4	2.1	2.4	1.9	1.9	7.9	13.6	2	4.6	4.2	5.8	5.2	14.9	14.6	14.7	20.8	50.3	3.1	1.9	1.3	2.3
GZNU20200706003	M	30.6	10.8	10.3	4.0	3.6	3.7	3.1	2.3	2.7	2.4	1.8	7.7	13.9	2.1	3.9	3.3	6.2	4.9	15.3	14.6	13.4	20.8	50.7	2.6	2.0	1.2	1.8
GZNU20200706004	M	32.3	11.2	11.1	4.3	3.3	4.3	3.4	2.0	2.8	2.2	2.0	8.3	14.5	2.3	4.3	4.5	6.1	4.7	16.5	15.4	15.1	22.5	54.4	3.3	2.1	1.2	2.0
GZNU20200706005	M	29.8	11.0	11.2	4.4	3.7	3.7	3.3	2.0	2.6	2.5	2.2	8.2	14.3	2	3.7	5.7	7.7	5.9	15.9	15.3	14.6	22.8	54.0	3.1	1.6	1.2	1.7
GZNU20200706006	M	30.4	11.2	10.6	4.2	3.3	3.7	3.3	1.7	2.5	2.7	1.7	7.3	13.2	1.9	4.3	4.0	5.6	4.4	15.6	15.1	13.7	21.1	51.8	3.2	2.1	1.7	1.9
GZNU20200706007	M	28.6	10.2	9.1	3.9	3.4	3.9	2.8	1.7	2.7	2.3	2.1	7.5	13.9	1.9	3.9	3.6	5.8	4.1	14.6	13.8	13.5	20.9	49.3	2.8	1.8	1.6	1.9
GZNU20200706008	M	31.4	10.8	10.4	4.9	3.6	3.2	3.3	2.1	2.9	2.2	2.0	8.23	14.7	1.7	4.4	4.3	6.3	4.5	16.2	16.3	13.8	22.1	54.6	2.9	2.3	1.4	1.8
GZNU20200706009	M	31.1	10.7	11.2	3.4	3.9	3.2	3.5	2.4	2.9	1.9	1.9	7.9	14.8	2.4	4.4	3.9	6.3	5.1	15.6	15.2	14.5	22.9	53.7	3.1	1.7	1.4	1.9
GZNU20200706012	M	33.3	11.5	10.9	4.3	3.6	3.8	3.3	2.2	2.6	1.9	2.1	8.1	14.6	2.1	4.3	4.9	6.1	4.8	16.2	16.1	15.3	23.5	55.8	3.4	1.8	1.2	2.1
GZNU20200706013	M	30.2	10.6	9.8	4.1	3.6	3.5	3.1	1.7	3.0	1.9	1.9	7.9	13.8	2.1	4.5	3.9	5.7	4.9	14.8	13.4	13.5	22.1	50.3	3.1	2.3	1.4	2.2
GZNU20200707001	M	31.2	11.4	10.4	4.1	3.9	3.9	3.4	2.3	2.9	2.4	1.9	8.5	15.2	2.0	4.5	4.5	6.9	4.9	16.9	15.5	15.1	23.1	55.5	3.4	1.7	1.4	2.0
GZNU20200707002	M	30.1	11.2	10.8	3.9	3.5	3.5	3.4	2.1	2.4	1.9	1.9	7.9	13.6	2	4.1	3.6	5.7	4.9	14.9	14.6	14.7	20.8	50.3	3.1	2.2	1.6	1.9
GZNU20200707003	M	31.8	11.4	11.1	4.5	3.5	3.7	3.3	2.3	2.9	2.3	1.9	8.5	14.9	2.4	4.7	3.8	6.5	4.8	16.6	15.6	15.1	22.4	54.6	3.2	2.5	1.5	2.6
GZNU20200706011	F	38.4	12.1	11.8	4.5	4.8	3.8	3.5	2.5	3.7	2.5	1.8	8.9	16.2	2.0	5.1	4.3	6.6	5.7	19.1	18.6	16.6	25.7	63.4	3.9	2.8	2.1	2.2
GZNU20200706004	F	39.2	13.2	11.7	4.3	4.3	3.7	3.9	2.5	3.9	2.3	1.9	9.5	16.3	1.9	4.8	5.9	7.5	6.4	19.2	19.5	17.8	26.3	65	3.6	2.9	2.3	2.4
GZNU20200706005	F	39.5	13.3	11.5	4.4	4.2	3.8	3.5	2.4	3.8	2.6	1.9	9.1	16.2	2.1	4.8	5.9	7.7	6.4	19.3	19.2	18.2	26.2	64.7	3.5	2.8	2.4	2.6
GZNU20200706006	F	40.2	14.5	13.2	5.1	5.2	4.5	4.8	3.4	3.9	2.5	2.2	9.8	17.4	2.0	4.8	6.0	7.7	6.8	20	19.9	18.9	26.8	66.7	3.8	2.9	2.9	2.9

Morphometric data were collected from 19 well-preserved adult specimens (voucher information given in Table 2). Measurements were recorded to the nearest 0.1 mm with digital calipers by Tao Luo following Fei et al. (2009). A total of 27 morphological features were measured in each well-preserved specimen. These following measurements were taken:

**ED** eye diameter (diameter of exposed portion of eyeball);

**FIL** first finger length;

**FIIL** second finger length;

**FIIIL** third finger length;

**FIVL** fourth finger length;

**FL** foot length (distance from distal end of tibia to the tip of the distal phalanx of toe IV);

**HDL** head length (from tip of snout to the articulation of the jaw);

**HDW** head width (head width at the commissure of the jaws);

**HLL** hindlimb length (distance from tip of fourth toe to vent);

**HND** hand length (from the proximal border of the outer palmar tubercle to the tip of digit III);

**IMTL** inner metatarsal tubercle length (taken as maximal length of inner metatarsal tubercle);

**IND** internasal distance (distance between nares);

**IOD** interorbital distance (minimum distance between upper eyelids);

**IPTL** inner palmar tubercle length (measured as maximal distance from proximal to distal ends of the inner palmar tubercle);

**LAHL** length of lower arm and hand (distance from the elbow to the distal end of finger IV);

**LW** lower arm width (maximum width of the lower arm);

**NED** nasal to eye distance (distance between the nasal and the anterior corner of the eye);

**OPTL** outer metacarpal tubercle length (measured as maximal diameter of outer metacarpal tubercle);

**SNT** snout length (from tip of snout to the anterior corner of the eye);

**SVL** snout-vent length (from tip of snout to posterior margin of vent);

**TD** tympanum diameter (horizontal diameter of tympanum);

**TED** tympanum to eye distance (distance from anterior edge of tympanum to posterior corner of eye);

**TFL** length of foot and tarsus (distance from the tibiotarsal articulation to the distal end of toe IV);

**THL** thigh length (distance from vent to knee);

**TL** tibia length (distance from knee to heel);

**TW** tibia width (maximum width of the tibia);

**UEW** upper eyelid width (greatest width of the upper eyelid margins measured perpendicular to the anterior-posterior axis).

To reduce allometric effects, all measurements were size-corrected with respect to SVL prior to morphometric analysis. Principal component analyses (PCAs) of size-corrected measurements and simple bivariate scatterplots were used to explore and characterize the morphometric differences between the new species and *P. leishanensis*. Mann–Whitney *U* tests were conducted to determine the significance of differences in morphometric characters between the new species and *P. leishanensis*. Mann–Whitney *U* tests also were used to test the significance of morphometric differences between males and females of the new species. All statistical analyses were performed using SPSS 21.0 (SPSS, Inc., Chicago, IL, USA), and differences were considered statistically significant at *P* < 0.05. Sex was determined based on male secondary sexual characters: the presence of a vocal sac and nuptial pads/spines (Fei and Ye 2016).

We compared the morphological characters of the new species with literature data for 59 other species in the *Panophrys* (Table 3). We also examined the type and/or topotype materials for *P. jiangi*, *P. liboensis*, *P. shuichengensis*, and *P. spinata* (see Appendix 1).

**Table 3.** References for morphological characters for congeners of the genus *Panophrys*.

ID	Species	Literature consulted
1	*Panophrys acuta* (Wang, Li & Jin, 2014)	Li et al. 2014
2	*Panophrys angka* (Wu, Suwannapoom, Poyarkov, Chen, Pawangkhanant, Xu, Jin, Murphy & Che, 2019)	Wu et al. 2019
3	*Panophrys anlongensis* (Li, Lu, Liu & Wang, 2020)	Li et al. 2020b
4	*Panophrys baishanzuensis* (Wu, Li, Liu, Wang & Wu, 2020)	Wu et al. 2020
5	*Panophrys baolongensis* (Ye, Fei & Xie, 2007)	Ye et al. 2007
6	*Panophrys binchuanensis* (Ye & Fei, 1995)	Ye and Fei 1995
7	*Panophrys binlingensis* (Jiang, Fei & Ye, 2009)	Fei et al. 2009
8	*Panophrys boettgeri* (Boulenger, 1899)	Fei et al. 2012
9	*Panophrys brachykolos* (Inger & Romer, 1961)	Inger and Romer 1961
10	*Panophrys caobangensis* (Nguyen, Pham, Nguyen, Luong & Ziegler, 2020)	Nguyen et al. 2020
11	*Panophrys caudoprocta* (Shen, 1994)	Fei et al. 2012
12	*Panophrys cheni* (Wang & Liu, 2014)	Wang et al. 2014
13	*Panophrys chishuiensis* (Xu, Li, Liu, Wei & Wang, 2020)	Xu et al. 2020
14	*Panophrys daiyunensis* Lyu, Wang & Wang, 2021	Lyu et al. 2021
15	*Panophrys daoji* Lyu, Zeng, Wang & Wang, 2021	Lyu et al. 2021
16	*Panophrys daweimontis* (Rao & Yang, 1997)	Fei et al. 2012
17	*Panophrys dongguanensis* (Wang & Wang, 2019)	Wang et al. 2019a
18	*Panophrys fansipanensis* (Tapley, Cutajar, Mahony, Nguyen, Dau, Luong, Le, Nguyen, Nguyen, Portway, Luong & Rowley, 2018)	Tapley et al. 2018
19	*Panophrys frigida* (Tapley, Cutajar, Nguyen, Portway, Mahony, Nguyen, Harding, Luong & Rowley, 2021)	Tapley et al. 2021
20	*Panophrys hoanglienensis* (Tapley, Cutajar, Mahony, Nguyen, Dau, Luong, Le, Nguyen, Nguyen, Portway, Luong & Rowley, 2018)	Tapley et al. 2018
21	*Panophrys huangshanensis* (Fei & Ye, 2005)	Fei et al. 2012
22	*Panophrys insularis* (Wang, Liu, Lyu, Zeng & Wang, 2017)	Wang et al. 2017a
23	*Panophrys jiangi* (Liu, Li, Wei, Xu, Cheng, Wang & Wu, 2020)	Liu et al. 2020
24	*Panophrys jingdongensis* (Fei & Ye, 1983)	Fei et al. 2012
25	*Panophrys jinggangensis* (Wang, 2012)	Wang et al. 2012
26	*Panophrys jiulianensis* (Wang, Zeng, Lyu & Wang, 2019)	Wang et al. 2019a
27	*Panophrys kuatunensis* (Pope, 1929)	Fei et al. 2012
28	*Panophrys leishanensis* (Li, Xu, Liu, Jiang, Wei & Wang, 2019 «2018»)	Li et al. 2018b
29	*Panophrys liboensis* (Zhang, Li, Xiao, Li, Pan, Wang, Zhang & Zhou, 2017)	Zhang et al. 2017
30	*Panophrys lini* (Wang & Yang, 2014)	Wang et al. 2014
31	*Panophrys lishuiensis* (Wang, Liu & Jiang, 2017)	Wang et al. 2017b
32	*Panophrys lushuiensis* (Shi, Li, Zhu, Jiang, Jiang & Wang, 2021)	Shi et al. 2021
33	*Panophrys minor* (Stejneger, 1926)	Wang et al. 2017a
34	*Panophrys mirabilis* (Lyu, Wang & Zhao, 2020)	Lyu et al. 2020
35	*Panophrys mufumontana* (J. Wang, Lyu & Y.Y. Wang, 2019)	Wang et al. 2019a
36	*Panophrys nankunensis* (Wang, Zeng &. Wang, 2019)	Wang et al. 2019a
37	*Panophrys nanlingensis* (Lyu, J. Wang, Liu & Y.Y. Wang, 2019)	Wang et al. 2019a
38	*Panophrys obesa* (Wang, Li & Zhao, 2014)	Li et al. 2014
39	*Panophrys ombrophila* (Messenger & Dahn, 2019)	Messenger et al. 2019
40	*Panophrys omeimontis* (Liu, 1950)	Fei et al. 2009
41	*Panophrys palpebralespinosa* (Bourret, 1937)	Fei et al. 2012
42	*Panophrys qianbeiensis* (Su, Shi, Wu, Li, Yao, Wang & Li, 2020)	Su et al. 2020
43	*Panophrys rubrimera* (Tapley, Cutajar, Mahony, Chung, Dau, Nguyen, Luong & Rowley, 2017)	Tapley et al. 2017
44	*Panophrys sangzhiensis* (Jiang, Ye & Fei, 2008)	Jiang et al. 2008
45	*Panophrys sanmingensis* Lyu & Wang, 2021	Lyu et al. 2021
46	*Panophrys shimentaina* (Lyu, Liu & Wang, 2020)	Lyu et al. 2021
47	*Panophrys shuichengensis* (Tian & Sun, 1995)	Tian et al. 2000
48	*Panophrys shunhuangensis* (Wang, Deng, Liu, Wu & Liu, 2019)	Wang et al. 2019b
49	*Panophrys spinata* (Liu & Hu, 1973)	Fei et al. 2012
50	*Panophrys tongboensis* Wang & Lyu, 2021	Lyu et al. 2021
51	*Panophrys tuberogranulata* (Shen, Mo & Li, 2010)	Mo et al. 2010
52	*Panophrys wugongensis* (J. Wang, Lyu & Y.Y. Wang, 2019)	Wang et al. 2019a
53	*Panophrys wuliangshanensis* (Ye & Fei, 1995)	Fei et al. 2012
54	*Panophrys wushanensis* (Ye & Fei, 1995)	Fei et al. 2012
55	*Panophrys xianjuensis* (Wang, Wu, Peng, Shi, Lu & Wu, 2020)	Wang et al. 2020
56	*Panophrys xiangnanensis* (Lyu, Zeng & Wang, 2020)	Lyu et al. 2021
57	*Panophrys yangmingensis* (Lyu, Zeng & Wang, 2020)	Lyu et al. 2020
58	*Panophrys yeae* (Shi, Zhang, Xie, Jiang, Liu, Ding, Luan & Wang, 2020)	Shi et al. 2020
59	*Panophrys zhoui* (Shi, Zhang, Xie, Jiang, Liu, Ding, Luan & Wang, 2020)	Shi et al. 2020

### Bioacoustics analyses

The advertisement calls of the new species were recorded from the holotype specimen (voucher number GZNU20200706010) in the field on 5 July 2020 at the Yueliangshan Nature Reserve, Congjiang County, Guizhou Province, China. The advertisement calls were recorded in a stream, using a digital sound recorder (TASCAM DR-40) at an ambient air temperature of 25 °C and 92% humidity. Sounds were recorded within 5 cm of the calling individual. The wave-format sound files were sampled at 44 kHz, with sampling depth 24 bits. Praat 6.1.16 (Boersma 2001) was used to obtain oscillograms, sonograms, and power spectra at a window length of 0.005 s. The ambient temperature at the type locality was measured using a digital hygrothermograph (ECOFIVE MS6508).

## Results

### Phylogenetic analyses and genetic divergence

**Figure 2.**
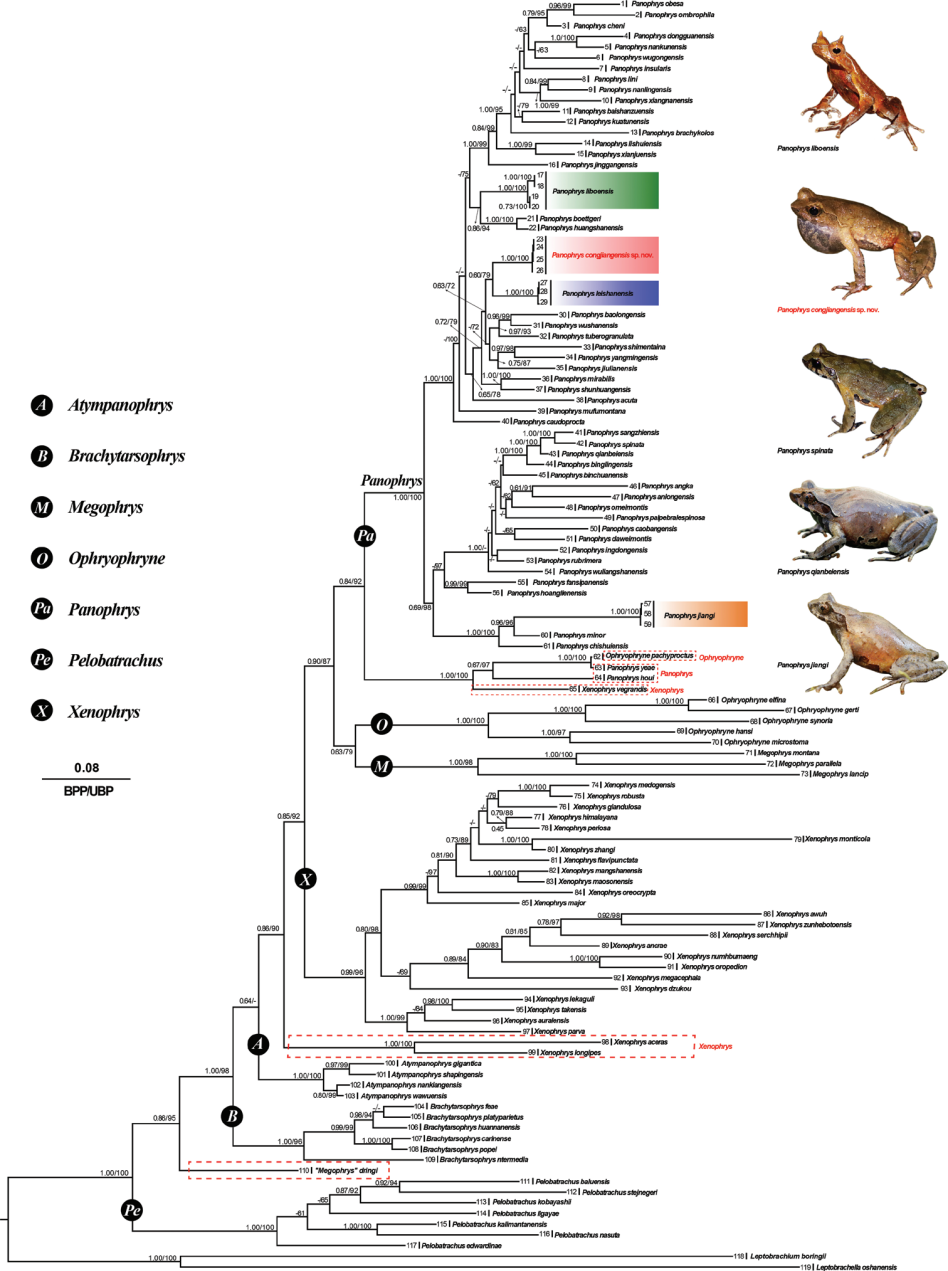
Phylogenetic tree based on mitochondrial 16S+COI genes. In this phylogenetic tree, ultrafast bootstrap supports (UFB) from ML analyses/Bayesian posterior probabilities (BPP) from BI analyses were noted beside nodes. The symbol “-” represents value below 0.60/60. Photos of new collections and 11 of 4 *Panophrys* species in Guizhou Province. The scale bar represents 0.08 nucleotide substitutions per site. The numbers at the tip of branches corresponds to the ID numbers in Table 1.

ML and BI phylogenies were constructed based on two concatenated mitochondrial gene sequences: 16S rRNA (548 bp) and COI (672 bp). The ML and BI topologies were largely identical (Fig. 2). *Panophrys* (except for *P. yeae* and *P. zhoui*) was strongly supported as monophyletic by both phylogenetic analyses. The phylogenetic trees also supported the monophyly of four of the seven genera of subfamily Megophryinae proposed in the revision of Li et al. (2020a): *Ophyrophryne*, *Atympanophrys*, *Brachytarsophrys*, *Panophrys* (except for *P. yeae* and *P. zhoui*), and *Pelobatrachus*; the monophyly of *Xenophrys* and *Ophyrophryne* was not supported. In both analyses, the new species formed a lower supported clade (0.59 in BI and 56% in ML) with *P. leishanensis*, *P. baolongensis*, *P. wushanensis*, *P. tuberogranulata*, *P. shimentaina*, *P. yangmingensis*, *P. jiulianensis*, *P. mirabilis*, *P. shunhuangensis*, and *P. acuta*. However, relationships among species in this clade were not well resolved except for the following well-supported sister relationships: *P. baolongensis* and *P. wushanensis*; *P. shimentaina* and *P. yangmingensis*; and *P. mirabilis* and *P. shunhuangensis*. The new species was recovered in a relatively poorly-supported sister relationship with *P. leishanensis* (0.60 in BI and 79% in ML; Fig. 2).

The smallest *p*-distance between this lineage and any other species of *Panophrys* was 1.2% in 16S rRNA (with *P. huangshanensis*) and 6.5% in COI (with *P. wushanensis*). These levels of divergence were similar to those between other pairs of recognized congeners. For example, the 16S rRNA*p*-distance was 1.2% between *P. leishanensis* and *P. huangshanensis*, 1.2% between *P. jingdongensis* and *P. binchuanensis*, while the COI*p*-distance was 5.9% between *P. lini* and *P. nanlingensis*, 3.6% between *P. spinata* and *P. sangzhiensis*, and 4.5% between *Brachytarsophrys carinense* and *B. popei* (Suppl. material 1: Table S1; Suppl. material 2: Table S2). These results suggested that this population, from the Yueliangshan Nature Reserve, Congjiang County, Guizhou Province, China, represented an independent evolutionary lineage.

### Species delimitation

The results of the *BEAST analysis for the alternative species model are provided in Table 4. Both SS and PS estimations based on 16S rRNA+COI datasets had the largest values for the 11 species taxonomy, indicating that it was supported in favor of the currently accepted 11 species model. In addition, the results of the maximum likelihood solution of the bPTP analysis supported 11 species taxonomy model (Appendix 1). Thus, the results of the BFD and bPTP analyses suggest support for treating the new species as a single valid species.

**Table 4.** The species delimitation results of new species and several closely related species in BF method.

Model	Species delimitation	MLE Path Sampling (PS)	MLE Stepping Stone (SS)	Rank	BF (PS)	BF (SS)
M1	11 species:	-4011.49	-4011.48	1	14.14	14.02
	AC+BA+JU+MI+SI+SU+TU+WU+YA+LE+CO					
M2	10 species:	-4018.56	-4018.49	2	–	–
	AC+BA+JU+MI+SI+SU+TU+WU+YA+{LE+CO}					

Each model represents a possible relationship of the new species to 10 closely related species. Abbreviation as: *P. acuta*: AC, *P. baolongensis*: BA, *P. jiulianensis*: JU, *P. mirabilis*: MI, *P. shimentaina*: SI, *P. shunhuangensis*: SU, *P. tuberogranulata*: TU, *P. wushanensis*: WU, *P. yangmingensis*: YA, *P. leishanensis*: LE, *P. congjiangensis* sp. nov.: CO.

### Morphological analyses

**Figure 3.**
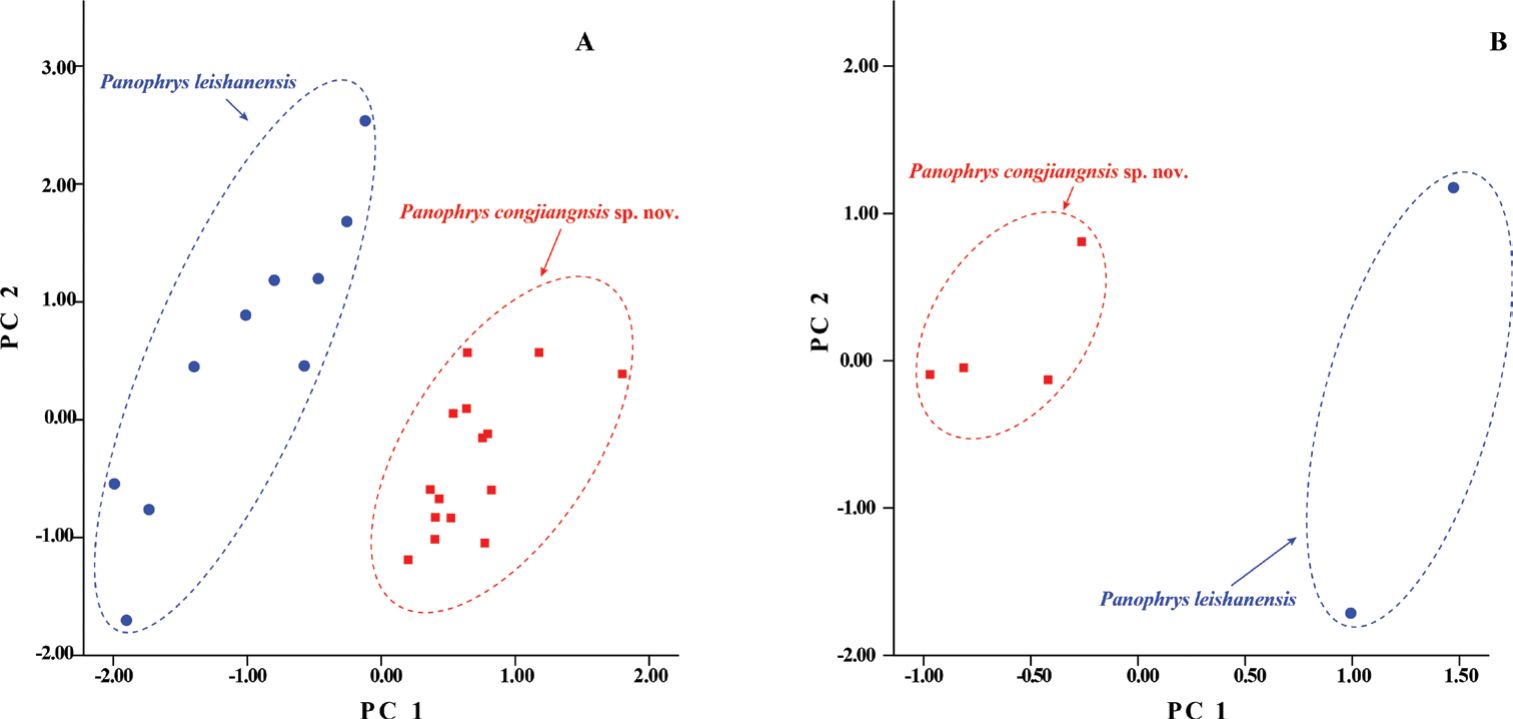
Plots of the first principal component (PC1) versus the second (PC2) for *Panophrys congjiangensis* sp. nov. and *P. leishanensis* from a principal component analysis **A** male **B** female.

The results of the Mann–Whitney *U* tests indicated that males of the new species differed significantly from *P. leishanensis* males based on several morphometric characters (all *p*-values < 0.05; Table 5). Using PCA, we extracted two and three principal component factors with Eigenvalues greater than two for males and females, respectively (Suppl. material 3: Table S3). The first two principal components explained 67.23% and 80.68% of the total variation in males and females, respectively. The variances in the data were mainly associated with limb and head characters, including TW, THL, HDL, LW, HDW, LAHL, HLL, FIIIL, FIL, FIIL, TFL, TL, IND, and IOD (Table 5). The characters of the new species were distinct from those of *P. leishanensis* on two-dimensional plots of PC1 and PC2 for both males and females (Fig. 3).

**Table 5.** Morphological comparison of *Panophrys congjiangensis* sp. nov. (*PC*) and *P. leishanensis* (*PL*). All units in mm. *P*-values are at 95% significance. Morphometric characters are explained in the methods section. C*M* and *CF* are the abbreviations of male and female from *Panophrys congjiangensis* sp. nov.

**Measurements**	***Panophrys congjiangensis* sp. nov.**				***Panophrys leishanensis^#^***			***P*-value from Mann-Whitney U test**		
	**Male (*N*=15)**		**Female (*N*=4)**		**Male (*N*=10)**		**Female (*N*=2)**	**Male**	**Female**	***CM vs.CF***
	**Range**	**Mean** ± **SD**	**Range**	**Mean** ± **SD**	**Range**	**Mean** ± **SD**	**Range**	***PC vs. PL***	***PC vs. PL***	
SVL	28.6–33.4	31.2 ± 1.4	38.4–40.2	39.3 ± 0.7	30.4–38.7	34.3 ± 2.7	42.3–42.3	0.000	0.133	0.003
HDL	10.2–11.5	11.0 ± 0.4	12.1–14.5	13.3 ± 1.0	9.1–11.0	10.1 ± 0.7	11.3–11.7	0.000	0.267	0.002
HDW	9.1–11.4	10.7 ± 0.6	11.5–13.2	12.1 ± 0.8	10.5–12.0	11.4 ± 0.5	12.1–12.4	0.014	0.133	0.003
SNT	3.4–4.9	4.2 ± 0.3	4.3–5.1	4.6 ± 0.4	3.6–4.5	4.2 ± 0.3	4.5–5.0	0.007	0.267	0.027
ED	3.3–3.9	3.6 ± 0.2	4.2–5.2	4.6 ± 0.5	3.3–4.3	3.9 ± 0.3	4.1–4.8	0.643	1.000	0.002
IOD	3.1–4.3	3.6 ± 0.3	3.7–4.5	4.0 ± 0.4	3.3–4.3	3.7 ± 0.3	3.9–4.2	0.062	0.267	0.084
IND	2.8–3.7	3.3 ± 0.2	3.5–4.8	3.9 ± 0.6	3.5–4.7	4.0 ± 0.4	4.1–4.3	0.392	0.267	0.011
TD	1.7–2.4	2.1 ± 0.2	2.4–3.4	2.7 ± 0.5	2.0–2.6	2.3 ± 0.2	2.5–2.8	0.461	1.000	0.003
UEW	2.4–3.0	2.7 ± 0.2	3.7–3.9	3.8 ± 0.1	/	/	/	/	/	0.002
NED	1.9–2.7	2.2 ± 0.3	2.3–2.6	2.5 ± 0.1	/	/	/	/	/	0.059
TED	1.7–2.2	1.9 ± 0.1	1.8–2.2	2.0 ± 0.2	/	/	/	/	/	0.750
HND	7.3–8.6	8.1 ± 0.4	8.9–9.8	9.3 ± 0.4	/	/	/	/	/	0.003
LAHL	13.2–15.2	14.3 ± 0.6	16.2–17.4	16.5 ± 0.6	14.4–16.3	15.3 ± 0.6	18.1–18.4	0.036	0.133	0.003
LW	1.7–2.4	2.1 ± 0.2	1.9–2.1	2.0 ± 0.1	2.7–3.9	3.2 ± 0.5	2.8–2.9	0.001	1.000	0.355
TL	14.6–17.5	15.9 ± 0.9	19.1–20	19.4 ± 0.4	16.2–18.6	17.5 ± 0.9	19.2–19.2	0.129	0.800	0.003
THL	13.4–17.8	15.3 ± 1.1	18.6–19.9	19.3 ± 0.5	14.4–16.8	15.4 ± 0.8	17.6–17.7	0.000	1.000	0.003
FL	13.4–15.7	14.6 ± 0.8	16.6–18.9	17.9 ± 1.0	14.9–17.3	15.9 ± 1.0	18.1–19.0	0.129	0.800	0.003
TFL	20.8–24.9	22.3 ± 1.2	25.7–26.8	26.3 ± 0.5	21.1–25.9	23.5 ± 0.5	27.5–27.9	0.004	1.000	0.003
HLL	49.3–60.2	53.5 ± 3.0	63.4–66.7	65.0 ± 1.4	50.3–60.2	54.2 ± 3.0	49.3–50.3	0.023	0.533	0.003
TW	2.6–3.5	3.1 ± 0.2	3.5–3.9	3.7 ± 0.2	3.6–4.7	4.2 ± 0.3	4.8–5.1	0.000	0.133	0.003
IPTL	1.6–2.5	2.0 ± 0.3	2.8–2.9	2.9 ± 0.1	/	/	/	/	/	0.003
OPTL	1.2–1.7	1.4 ± 0.2	2.1–2.9	2.4 ± 0.3	/	/	/	/	/	0.002
IMTL	1.7–2.6	2.0 ± 0.2	2.2–2.9	2.5 ± 0.3	/	/	/	/	/	0.014
FIL	3.7–4.7	4.3 ± 0.3	4.8–5.1	4.9 ± 0.2	3.2–3.9	3.5 ± 0.2	4.0–4.3	0.000	0.133	0.003
FIIL	3.3–5.7	4.2 ± 0.6	4.3–6	5.5 ± 0.8	2.8–3.5	3.2 ± 0.3	3.8–4.1	0.000	0.133	0.011
FIIIL	5.6–7.7	6.2 ± 0.5	6.6–7.7	7.4 ± 0.5	4.2–5.4	4.8 ± 0.4	5.4–5.8	0.000	0.133	0.009
FIVL	4.1–5.9	4.9 ± 0.4	5.7–6.8	6.3 ± 0.5	3.4–4.1	3.7 ± 0.2	4.2–4.3	0.000	0.133	0.004

Note : ^#^ Morphological data from Li et al .(2018b).

### Taxonomic account

#### Panophrys congjiangensis sp. nov.

Taxon classificationAnimaliaHymenostomatidaOphryoglenidae

AF664652-FBED-57D2-A8EC-DBD12779CA19

http://zoobank.org/B433A7B8-2C23-4EC7-8C94-BC7FECF8B584

**Figure 4.**
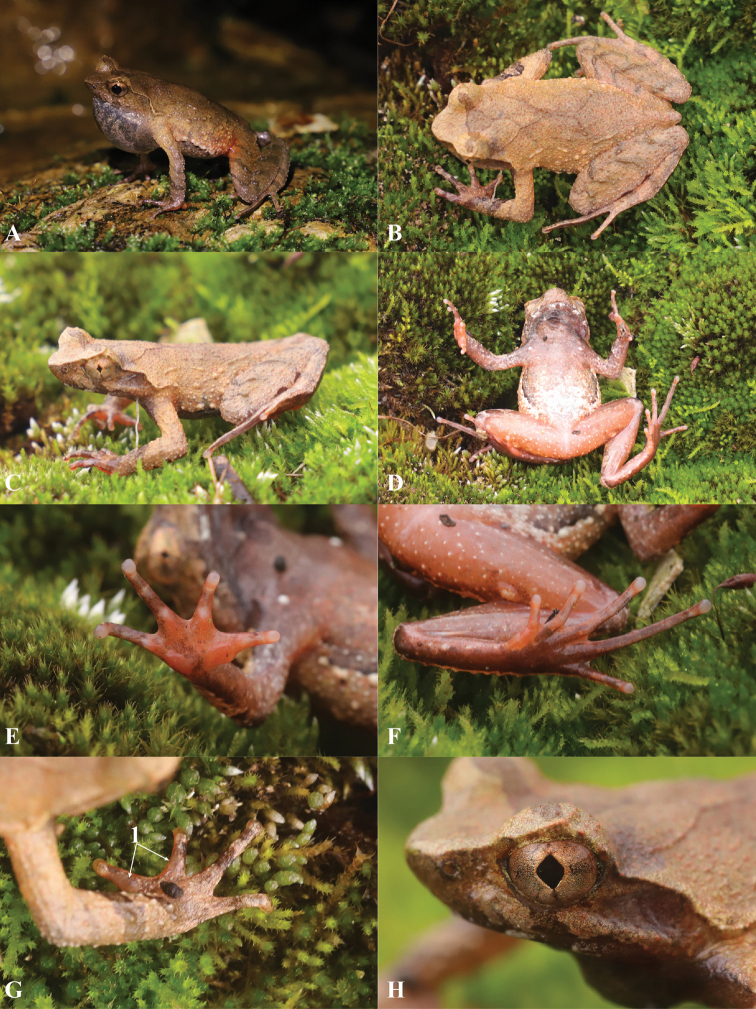
Morphological features of the live adult male holotype GZNU20200706010 of *Panophrys congjiangensis* sp. nov. **A** single subgular vocal sac **B** dorsal view **C** dorsolateral view **D** ventral view **E** ventral view of hand **F** ventral view of foot **G** dorsal view of hand (1 indicates villiform gray-black nuptial spines) **H** iris. A was photographed at about 9 p.m., and **B** to **H** during the day, respectively.

**Figure 5.**
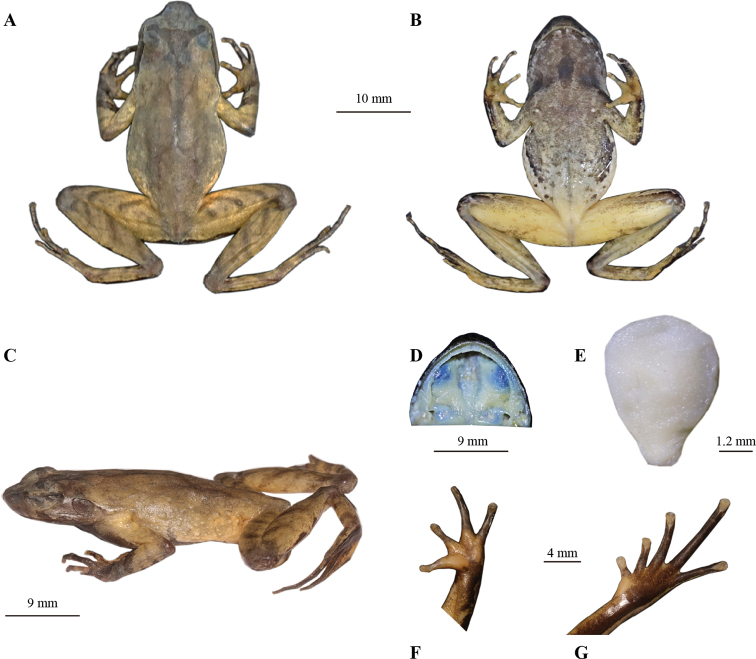
Morphological features of the preserved adult male holotype GZNU20200706010 of *Panophrys congjiangensis* sp. nov. **A** dorsal view **B** ventral view **C** lateral view **D** view of oral cavity **E** tongue **F** ventral view of hand **G** ventral view of foot.

**Figure 6.**
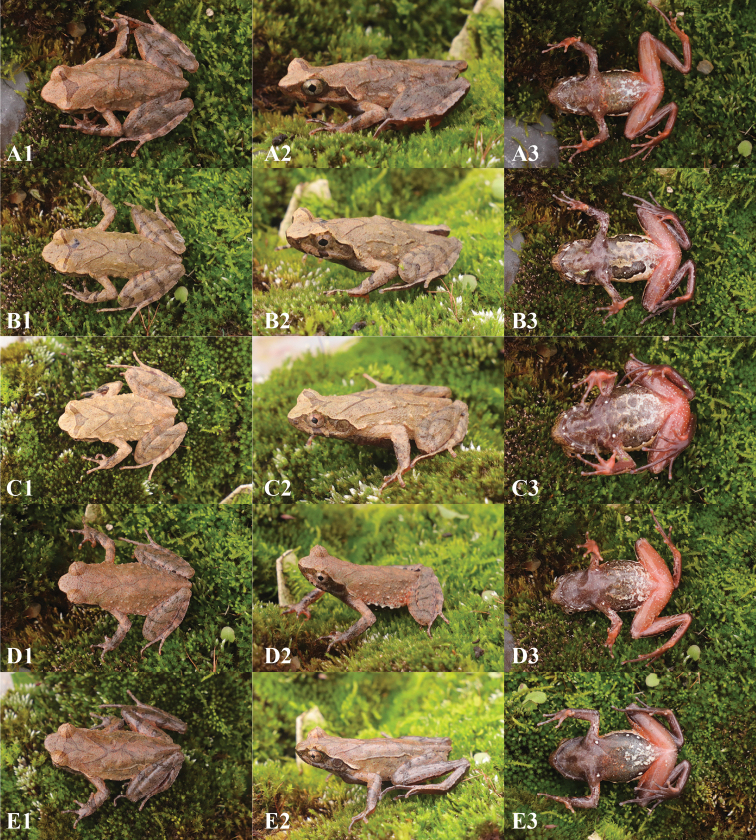
Paratypes of *Panophrys congjiangensis* sp. nov. in life **A** GZNU20200706007, adult male **B** GZNU20200706008, adult male **C** GZNU20200706009, adult male **D** GZNU2020706013, adult male **E** GZNU20200706012, adult female. So, the images were all taken at 8 am.

Table 2 , Figs 4 , 5 , 6

##### Type material.

***Holotype*.** GZNU20200706010 (Figs 4, 5), adult male, collected by Tao Luo on 6 May 2020 in the Yueliangshan Nature Reserve, Congjiang County, Guizhou Province, China (25.614417°N, 108.410076°E; ca. 730 m a.s.l.).

***Paratypes*.** Nineteen adult specimens (15 males and 4 females) from the same locality. Eleven males (GZNU20200706001–06009, GZNU20200706012–06013) collected with the holotype on 6 July 2020 by Tao Luo, Xueli Lu, and Weifeng Wang. One female (GZNU20200706011) collected with the holotype by Tao Luo. Three males (GZNU200707001–07003) collected on 7 July 2020 by Tao Luo. Three females (GZNU20200706004, GZNU20200706005, and GZNU20200706006) collected on 7 July 2020 by Tao Luo.

##### Etymology.

The specific epithet “congjiangensis” refers to the holotype locality, which is Yueliangshan Nature Reserve, located in Congjiang County, Guizhou Province, China. We propose the English common name “Congjiang Horned Toad” and the Chinese common name “Cong Jiang Jiao Chan (从江角蟾)”.

##### Differential diagnosis.

*Panophrys congjiangensis* sp. nov. is assigned to the genus *Panophrys* based on molecular phylogenetic analyses and the following characteristics, which are diagnostic for this genus: (1) snout shield-like; (2) snout projecting beyond the lower jaw; (3) tympanum distinct (4) canthus rostralis distinct; (5) chest gland small and round, closer to axilla than to midventral line; (6) femoral gland on rear of thigh; (7) vertical pupils (Fei et al. 2006; Fei and Ye 2016; Su et al. 2020).

*Panophrys congjiangensis* sp. nov. is distinguished from its congeners by a combination of the following characters: (1) medium body size (SVL: 28.6–33.4 mm in males and 38.4–40.2 mm in females); (2) single small horn-like tubercle at edge of each upper eyelid; (3) tympanum distinctly visible (TD/ED ratio 0.47–0.66); (4) vomerine teeth absent; (5) tongue not notched behind; (6) a narrow and unobvious lateral fringe on toes; (7) relative finger lengths II < I < V < III; (8) rudimentary webs on toes; (9) hindlimbs slender, heels overlapping when thighs are positioned at right angles to body; (10) two metacarpal tubercles on palm, with inner metatarsal tubercle long and oval-shaped; (11) tibiotarsal articulation reaching nostril when leg is stretched forward; (12) dorsal skin rough, with numerous orange-red granules, ventral surface smooth; (13) single internal subgular vocal sac present in males; (14) in breeding males, weak gray-black nuptial pads with black nuptial spines present on dorsal surfaces of bases of first and second fingers.

##### Description of holotype.

GZNU20200706010 (Figs 4, 5), adult male. Medium body size, SVL 33.4 mm; head length slightly larger than head width (HDL/HDW ratio 1.02); snout short, rounded and projecting beyond the lower jaw in dorsal view, longer than eye diameter (SNT/ED ratio 1.11); nostril rounded, distinct, and closer to the tip of the snout than to the eye (SNT/NED ratio 1.83); internasal distance greater than interorbital distance (IND/IOD ratio 1.19); internasal distance greater than upper eyelid width (IND/UEW ratio 1.28); region vertical and concave; canthus rostralis well-developed; top of head slightly concave in dorsal view; a small horn-like tubercle at the edge of the upper eyelid; eyes large, slightly protuberant in dorsal view, eye diameter 34% of head length, pupils vertical (Fig. 4H); tympanum distinct, tympanum diameter less than eye diameter (TD/ED ratio 0.63); vomerine ridges and vomerine teeth absent; tongue is melon seed-shaped and not notched behind (Fig. 5E).

Forelimbs slender and comparatively short, the length of lower arm and hand 44.01% of SVL; fingers slender, relative finger lengths: II < I < IV < III; tips of fingers slightly dilated, round, without lateral fringes; one distinct subarticular tubercle at the base of each finger; two metacarpal tubercles on the palm; prominent, the outer one long and thin, the inner one oval-shaped, inner metacarpal tubercles longer than outer metacarpal tubercles (IPTL/OPT ratio 1.13).

Hindlimbs slender (HLL/SVL ratio 1.80); heels slightly overlapping when thighs are positioned at right angles to the body; tibiotarsal articulation reaching the nostril when leg stretched forward; foot length less than tibia length (FL/TL ratio 0.90); relative toe lengths I < II < V < III < IV; tips of toes round and slightly dilated; toes with narrow and unobvious lateral fringes and rudiment webs; one subarticular tubercle at the base of each toe; inner metatarsal tubercle long oval-shaped and the outer one absent.

Dorsal skin rough with numerous orange-red granules; several large warts scattered on flanks and dorsal limbs; several tubercles on upper eyelid, including a small horn-like prominent tubercle on the edge (Fig. 4H); supratympanic fold distinct; tubercles on the dorsum forming a discontinuous X-shaped ridge, the V-shaped ridges disconnected; two discontinuous dorsolateral parallel ridges on either side of the X-shaped ridges; an inverted triangular brown speckle between two upper eyelids; four transverse skin ridges on the dorsal shank and thigh; ventral surface smooth; chest with small, round glands, closer to the axilla than to midventral line; femoral glands on rear of thigh; numerous white granules on ventral surface of thigh; posterior end of body distinctly protruding, forming an arc-shaped swelling above anal region.

##### Coloration of holotype in life

**(Fig. 4).** Dorsal surfaces of head and trunk brownish gray; triangular marking with light edge between eyes; dark X-shaped marking with light edge on central dorsum; supratympanic fold light brown; four dark brown transverse bands on dorsal surfaces of thigh and shank; 2–4 dark brown and white vertical bars on lower and upper lip; dark vertical band below eye; iris copper-brown; throat and anterior chest light purple-brown; belly light orange-red with large white blotch and small grey blotch in belly center, and small white blotches and large black patches on belly sides, forming a discontinuous line; ventral surfaces of forelimbs purplish brown; some white spots on the ventral surfaces of hindlimbs; palms orange-red with a small black-brown blotch; ventral surfaces of first and second toes orange-red, ventral surfaces of remaining three toes black-brown; soles black-brown; tips of digits grey-white; pectoral and femoral glands white.

##### Preserved holotype coloration

**(Fig. 5).** After preservation in ethanol, dorsal surfaces light brownish grey; dorsal surface of head dark gray; X-shaped ridges on dorsum indistinct and transverse bands on limbs and digits distinct, coloration lighter; throat dark black-brown; chest light black-brown; belly light gray-white with large black-brown blotches on sides and a small gray-brown blotch in center; posterior ventral body surface, inner thigh, and upper part of tibia milky yellow; palms and metatarsal tubercle milky yellow with a small gray-brown blotch; ventral surfaces of soles and toes dark black-brown; inner metatarsal tubercle milky yellow.

##### Variations.

Measurements of the type series are shown in Tables 2, 4. Females (SVL 39.3 ± 0.7 mm, *N* = 4) had larger bodies than males (SVL 31.2 ± 1.4 mm, *N* = 15). In life, the diagnostic morphological characters of all paratypes were identical to those of the holotype. However, coloration and stripe patterns differed among individuals (Fig. 6). For example, male GZNU20200706007 (Fig. 6A) had a brown-black back and a black-brown belly with some large white patches, as well as two V-shaped markings that were virtually connected. This specimen also had warts on both sides of the body, forming a transverse skin ridge that almost connected to the second V-shaped marking. In contrast, male GZNU20200706008 (Fig. 6B) had a large black spot between the upper eyelids. The throat and anterior belly of this specimen were purple–brownish, while the belly was light milky yellow, with two large black blotches and a small white blotch on the body sides. In specimens GZNU20200706009 and GZNU20200706012 (Fig. 6C, E), the warts on both sides of the body formed transverse skin ridges connected to the second V-shaped marking and extending behind the tympanum; three white small blotches were present on the body sides. In specimens GZNU20200706013 and GZNU20200706012 (Fig. 6D, E), the back was light reddish brown.

##### Advertisement call.

**Figure 7.**
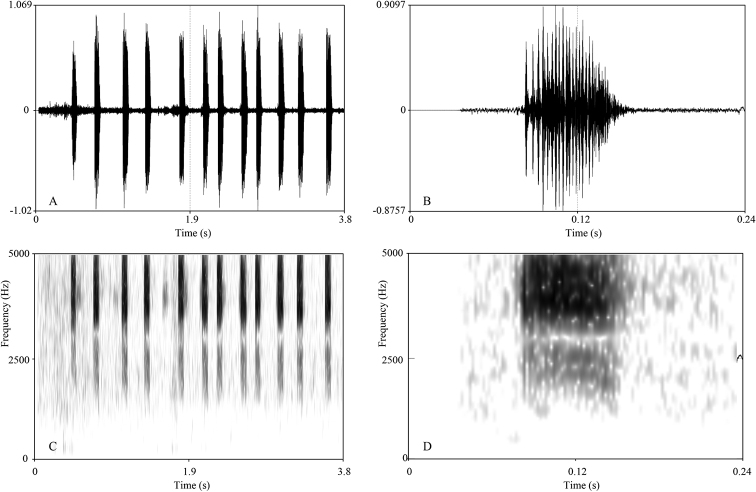
Visualization of advertisement calls of *Panophrys congjiangensis* sp. nov. **A** waveform showing 12 notes of one call **B** waveform showing one note **C** sonogram showing 12 notes of one call **D** sonogram showing one note.

The call description is based on recordings of the holotype GZNU 20200706010 (Fig. 7) from the bamboo forest near the streamlet. The ambient air temperature during the recording was 25.3 °C. Each call contains 9–14 syllables (mean 11.60 ± 2.07, *N* = 5). The call consists of a few strophes, each 2.41–3.43 s in duration (mean 2.75 ± 0.46, *N* = 4). Each syllable has a duration of 0.05–0.09 s (mean 0.07 ± 0.06, *N* = 58). The interval between syllables has a duration of 0.10–0.31 s (mean 0.167 ± 0.042, *N* = 53).

##### Sexual dimorphism.

Adult males (SVL 28.6–33.4 mm) smaller than adult females (SVL 38.4–40.2 mm). Adult males with single internal subgular vocal sac (Fig. 4A). Breeding males with grey-black nuptial pads with obvious black nuptial spines on dorsal surfaces of bases of first and second fingers.

##### Comparisons.

Comparative data of *Panophrys congjiangensis* sp. nov. with 59 recognized congeners of *Panophrys* are given in Suppl. material 4: Table S4.

By having small body size, SVL 30.4–34.1 mm in males, *Panophrys congjiangensis* sp. nov. differs from *P. baolongensis* (42.0–45.0 in males), *P. binlingensis* (45.1–51.0 in males), *P. boettgeri* (34.5–37.8 in males), *P. caobangensis* (34.9–38.9 in males), *P. caudoprocta* (81.3 in single male), *P. hoanglienensis* (37.4–47.6 in males), *P. huangshanensis* (36.0–41.6 in males), *P. insularis* (36.8–41.2 in males), *P. jingdongensis* (53.0–56.5 in males), *P. mirabilis* (55.8–61.4 in males), *P. obesa* (35.6 in single male), *P. palpebralespinosa* (36.2–38.0 in males), *P. sangzhiensis* (54.7 in single male), and *P. xiangnanensis* (38.6–42.0 in males). By having larger body size, SVL 30.4–34.1 mm in males, *Panophrys congjiangensis* sp. nov. differs from *P. cheni* (26.2–29.5 in males), *P. daiyunensis* (27.6–28.7 in males), *P. kuatunensis* (26.2–29.6 in males), *P. sanmingensis* (27.0–29.5 in males), *P. yeae* (23 in single male), and *P. zhoui* (23.8–29.1 in males). By having small body size, SVL 38.9–40.2 mm in females, *Panophrys congjiangensis* sp. nov. differs from *P. fansipanensis* (41.7–42.5 in females), *P. minor* (42.0–48.2 in females), *P. tuberogranulata* (50.5 in single female), *P. wuliangshanensis* (41.3 in single female), *P. xianjuensis* (41.6 in single female), and *P. yangmingensis* (45.2 in single female).

Nine *Panophrys* species were previously recorded from the Guizhou Province, namely *P. anlongensis*, *P. chishuiensis*, *P. jiangi*, *P. leishanensis*, *P. liboensis*, *P. omeimontis*, *P. shuichengensis*, *P. spinata*, and *P. qianbeiensis. Panophrys congjiangensis* sp. nov. differs from *P. anlongensis* by having small body size, SVL 30.4–34.1 mm in males and 38.9–40.2 mm in females (vs. 40.0–45.5 mm in males and 48.9–51.2 in females), tibiotarsal articulation reaching the nostril when leg stretched forward (vs. reaching to the level of mid-eye). *Panophrys congjiangensis* sp. nov. differs from *P. chishuiensis* by having small body size, SVL 30.4–34.1 mm in males and 38.9–40.2 mm in females (vs. 43.4–44.1 mm in males and 44.8–49.8 in females), rudimentary webs on toes (vs. lacking webs), subarticular tubercles present on each toes (vs. absent), tibiotarsal articulation reaching the nostril when leg stretched forward (vs. reaching the level between tympanum and eye). *Panophrys congjiangensis* sp. nov. differs from *P. jiangi* by having slightly small body size, SVL 30.4–34.1 mm in males (vs. 34.4–39.2 mm in males), relative finger lengths are II < I < V < III (vs. I < II < V < III), tibiotarsal articulation reaching the nostril when leg stretched forward (vs. reaching forward to the region between tympanum and eye). *Panophrys congjiangensis* sp. nov. differs from *P. liboensis*, *P. omeimontis*, *P. qianbeiensis*, *P. shuichengensis*, and *P. spinata* by having small body size, SVL 30.4–34.1 mm in males and 38.9–40.2 mm in females (vs. SVL>40 mm in males in *P. liboensis*, *P. omeimontis*, *P. qianbeiensis*, *P. shuichengensis*, and *P. spinata*; vs. SVL>50 mm in females in *P. liboensis*, *P. omeimontis*, *P. shuichengensis*, and *P. spinata*), small horn-like tubercle at the edge of each upper eyelid (vs. slightly large in *P. liboensis* and *P. shuichengensis*; absence in *P. qianbeiensis*), absence of vomerine teeth (vs. present in *P. liboensis*, *P. omeimontis*, and *P. qianbeiensis*), tongue not notched behind (vs. notched in *P. liboensis*, *P. omeimontis*, *P. qianbeiensis*, *P. shuichengensis*, and *P. spinata*), lateral fringes on toes narrow and unobvious (vs. wide in *P. liboensis*, *P. qianbeiensis*, *P. shuichengensis*, and *P. spinata*), rudimentary webs on toes (vs. more than one-fourth webs in *P. qianbeiensis*, *P. shuichengensis*, and *P. spinata*), subarticular tubercles present on each toes (vs. absent in *P. liboensis* and *P. shuichengensis*), tibiotarsal articulation reaching the nostril when leg stretched forward (vs. reaching to ocular region in *P. liboensis*, *P. omeimontis*, *P. shuichengensis*, and *P. spinata*; reaching to the level between tympanum and eye in *P. qianbeiensis*). *Panophrys congjiangensis* sp. nov. differs from *P. leishanensis* by having slightly small body size, SVL 38.9–40.2 mm in females (vs. 42.3 in single female), having narrow and unobvious lateral fringes on toes (vs. lacking), tibiotarsal articulation reaching the nostril when leg stretched forward (vs. reaching between tympanum to eye). The mean SVL of male *Panophrys congjiangensis* sp. nov. was significantly greater than that of *P. leishanensis*. In addition, the ratios of HDL, HDW, SNT, LAHL, LW, THL, TFL, HLL, and TW to SVL were all significantly greater in male *Panophrys congjiangensis* than in male *P. leishanensis* (all *p*-values < 0.05; Table 4). *Panophrys congjiangensis* sp. nov. also differs from *P. leishanensis* by having one call 9–14 syllables (vs. calls of *P. leishanensis*, which are 12–14 syllables long), shorter call intervals between syllables (0.167 ± 0.042 s, *N* = 53 in the new species vs. 0.409 ± 0.075 s, *N* = 36 in *P. leishanensis*), and shorter call syllables (0.07 ± 0.06 s, *N* = 58 in the new species vs. 0.105 ± 0.003 s, *N* = 37 in *P. leishanensis*).

From the remaining 24 species occurring in *Panophrys*, *Panophrys congjiangensis* sp. nov. can be distinguished by the absence of vomerine teeth (vs. present in *P. daweimontis*, *P. dongguanensis*, *P. frigida*, *P. jinggangensis*, *P. jiulianensis*, *P. nankunensis*, *P. nanlingensis*, *P. rubrimera*, *P. shimentaina*, and *P. tongboensis*), by the unnotched tongue (vs. tongue notched in *P. binchuanensis*, *P. cheni*, *P. kuatunensis*, and *P. lushuiensis*), by the small horn-like tubercle at edge of upper eyelid (vs. slightly large in *P. acuta*), by the absence of lateral fringes on toes (vs. lacking in *P. angka*, *P. brachykolos*, *P. lishuiensis*, *P. ombrophila*, *P. shunhuangensis*, and *P. wugongensis*; vs. wide in *P. lini*; vs. lacking in males in *P. wushanensis*, wide in females in *P. wushanensis*), by the subarticular tubercles present (vs. absent in *P. baishanzuensis* and *P. mufumontana*), tibiotarsal articulation reaching the nostril when leg stretched forward (vs. reaching to ocular region in *P. acuta*, *P. baishanzuensis*, *P. binchuanensis*, *P. jiulianensis*, *P. lini*, *P. nanlingensis*, *P. ombrophila*, and *P. wushanensis*; vs. reaching to the level between tympanum and eye in *P. angka*, *P. dongguanensis*, *P. kuatunensis*, *P. lishuiensis*, and *P. nankunensis*; vs. reaching to the level between eye and snout in *P. cheni*, *P. daweimontis*, and *P. shunhuangensis*; vs. reaching to the level behind the eye in *P. brachykolos*, *P. mufumontana*, *P. shimentaina*, and *P. wugongensis*; vs. reaching to the level at center of tympanum *P. daoji*).

##### Distribution and ecology.

*Panophrys congjiangensis* sp. nov. is only known from the type locality, Yueliangshan Nature Reserve, Congjiang County, Guizhou Province, China at elevations of 1142–1206 m. Individuals of the new species were frequently found in bamboo forests, grasses, and shrubberies near streams. Plants in the type locality predominantly fall into the families Urticaceae, Gramineae, Cyperaceae, Rosaceae, Dryopteridaceae, Polygonaceae, Aquifoliaceae, and Fagaceae. In the Yueliang Mountains, *Panophrys congjiangensis* sp. nov. is sympatric with *Pachytriton inexpectatus* Nishikawa, Jiang, Matsui & Mo, 2010; *Amolops sinensis* Lyu, Wang & Wang, 2019; *Nidirana leishanensis* Li, Wei, Xu, Cui, Fei, Jiang, Liu & Wang, 2019; *Hylarana latouchii* (Boulenger, 1899); *Quasipaa boulengeri* (Günther, 1889); *Hyla annectans* (Jerdon, 1870); *Opisthotropis zhaoermii* Ren, Wang, Jiang, Guo & Li, 2017; *Trimeresurus stejnegeri* (Schmidt, 1925); and *Rhabdophis tigrinus* (Boie, 1826). These species were often found in the same streams as *Panophrys congjiangensis* sp. nov.

## Discussion

Phylogenetic analyses based on two mitochondrial genes suggested that the specimens collected in this study fell into the *Panophrys*, but were distinct from all previously described species in this genus. Genetic distances between *Panophrys congjiangensis* sp. nov. and its sister species *P. leishanensis* were 3.0% for 16S rRNA and 8.4% for COI, within the ranges expected for interspecific divergences in amphibians (Fouquet et al. 2007; Che et al. 2012). Indeed, other species have been distinguished and recognized based on much lower genetic distances. For example, the *p*-distance is 1.2% between *P. angka* and *P. anlongensis* for 16S rRNA, and 3.6% between *P. sangzhiensis* and *P. spinata* for COI (Suppl. material 1: Table S1; Suppl. material 1: Table S2). *Panophrys congjiangensis* sp. nov. is morphologically similar to *P. leishanensis*, but *Panophrys congjiangensis* sp. nov. is smaller, has a narrow and unobvious lateral fringe on the toes, and the tibiotarsal articulation of the hindlimb reaches the nostril when the leg is adpressed and stretched forward. The two species can also be distinguished based on bioacoustics characters: the call of *Panophrys congjiangensis* sp. nov. had fewer syllables than that of *P. leishanensis*, and the call intervals were shorter. Without phylogenetic, morphological, and bioacoustics data, it is difficult to determine the taxonomic status of new species. In this study, these multiple pieces of evidence supported the validity of *Panophrys congjiangensis* sp. nov. The new species described in this study increases the number of species assigned to *Panophrys* to 60, with 56 recorded from China (Fei and Ye 2016; AmphibiaChina 2020; Frost 2021).

Climatic fluctuations, habitat heterogeneity, habitat diversity, and the dynamics of montane forests may play important roles in driving diversification in the *Panophrys* (Chen et al. 2017; Liu et al. 2018). These factors may have led to the development of complex phenotypes in this genus. Recent studies have revealed high levels of species diversity in the *Panophrys* (Frost 2021). However, *Panophrys congjiangensis* sp. nov. does not belong to any of the clades identified by Chen et al. (2017) and Liu et al. (2018), suggesting that *Panophrys* diversity may remain severely underestimated, even where *Panophrys* species are sympatric ally distributed (Li et al. 2018; Lyu et al. 2020; Su et al. 2020). Until recently, it was difficult to perform taxonomic and phylogenetic studies of the *Panophrys* because many species in this genus are morphologically similar and have sympatric distributions; the many possible cryptic species in the *Panophrys* may have hindered our understanding of diversity in this genus (Chen et al. 2017; Liu et al. 2018; Li et al. 2018; Wang et al. 2019a, b; Mahony et al. 2020; Lyu et al. 2020; Liu et al. 2020; Xu et al. 2020). The high species diversity, strong forest dependence, and sympatric distributions in the *Panophrys* indicate that speciation patterns, niche differentiation, and coexistence mechanisms in this genus require further study.

Biodiversity conservation in southwestern China is a priority of the Chinese government (Ministry of Environmental Protection 2015). Biodiversity conservation programs in this region play an important role in maintaining the stability of mountain ecosystems as well as protecting biodiversity (Körner and Spehn 2002; Tang et al. 2006). Mountain ecosystems are characterized by high biodiversity, with species tending to exhibit a wide range of evolutionary adaptations (McCain and Colwell 2011; Elsen and Tingley 2015). Mountain ecosystems also serve as sanctuaries for many endemic and threatened species, and thus play a major role in the maintenance of biodiversity (Favre et al. 2016). Mountains ecosystems provide key ecological service functions and provide important natural resources that are utilized by local human populations (Körner and Spehn 2002; Grêt-Regamey et al. 2012). Thus, mountain species face a higher risk of extinction due to their limited range, unique environmental adaptations, and geographic isolation, rendering mountain taxa among the most likely to be threatened by climate change.

In the past three years alone, 11 new amphibian species have been described from Guizhou Province, China (Zhang et al. 2017; Li et al. 2018a, b; Li et al. 2019a, b; Lyu et al. 2019b; Wang et al. 2019c; Wei et al. 2020; Luo et al. 2020; Liu et al. 2020; Su et al. 2020). The discovery of these new species suggests that amphibian species diversity in this region is severely underestimated. In the context of global warming, there is an urgent need for a comprehensive, systematic, and in-depth survey of the impacts of climate change on terrestrial vertebrates to provide a basis for scientific decisions regarding amphibian conservation (IPCC 2014).

## Supplementary Material

XML Treatment for Panophrys congjiangensis

## Appendix 1

**Specimens examined**

***Panophrys jiangi*** (*N*=3): China: Guizhou Province: Suiyang County: Kuankuosui National Nature Reserve (type locality): GZNU20070712001. China: Guizhou: Suiyang: Huoqiuba Nature Reserve (topotype locality): GZNU20180606020–606022.

***Panophrys liboensis*** (*N*=5): China: Guizhou Province: Libo County (type locality): GNUG20150813001, 408002, 408004, 408006–408008.

***Panophrys shuichengensis*** (*N*=7): China: Guizhou Province: Shuicheng County (type locality): 944001, 98001, 98002, 945005, 2007030, 2007031, 2007032.

***Panophrys spinata*** (*N*=6): China: Guizhou Province: Dafang County (topotype locality): GZNU201707015011–606016.

## Supplementary materials

Supplementary material 1 Table S1. Uncorrected *p*-distance between Megophryinae species based on 16s rRNA sequences Data type molecular data https://binary.pensoft.net/file/559432This dataset is made available under the Open Database License (http://opendatacommons.org/licenses/odbl/1.0/). The Open Database License (ODbL) is a license agreement intended to allow users to freely share, modify, and use this Dataset while maintaining this same freedom for others, provided that the original source and author(s) are credited.Tao Luo, Yali Wang, Siwei Wang, Xueli Lu, Weifeng Wang, Huaiqing Deng, Jiang Zhou

Supplementary material 2 Table S2. Uncorrected *p*-distance between Megophryinae species based on COI gene sequences Data type molecular data https://binary.pensoft.net/file/559433This dataset is made available under the Open Database License (http://opendatacommons.org/licenses/odbl/1.0/). The Open Database License (ODbL) is a license agreement intended to allow users to freely share, modify, and use this Dataset while maintaining this same freedom for others, provided that the original source and author(s) are credited.Tao Luo, Yali Wang, Siwei Wang, Xueli Lu, Weifeng Wang, Huaiqing Deng, Jiang Zhou

Supplementary material 3 Table S3. Variable loadings for principal components with Eigenvalues greater than 2, from morphometric characters corrected by SVL Data type statistical data https://binary.pensoft.net/file/559434This dataset is made available under the Open Database License (http://opendatacommons.org/licenses/odbl/1.0/). The Open Database License (ODbL) is a license agreement intended to allow users to freely share, modify, and use this Dataset while maintaining this same freedom for others, provided that the original source and author(s) are credited.Tao Luo, Yali Wang, Siwei Wang, Xueli Lu, Weifeng Wang, Huaiqing Deng, Jiang Zhou

Supplementary material 4 Table S4. Diagnostic characters separating all 59 species of the *Panophrys* Data type species data Explanation note Data modified from Wang et al. 2020; Li et al. 2020; Lyu et al. 2020; Nguyen et al. 2020. https://binary.pensoft.net/file/559435This dataset is made available under the Open Database License (http://opendatacommons.org/licenses/odbl/1.0/). The Open Database License (ODbL) is a license agreement intended to allow users to freely share, modify, and use this Dataset while maintaining this same freedom for others, provided that the original source and author(s) are credited.Tao Luo, Yali Wang, Siwei Wang, Xueli Lu, Weifeng Wang, Huaiqing Deng, Jiang Zhou
