# Supplementary material for: A species of the genus Panophrys (Anura, Megophryidae) from southeastern Guizhou Province, China
Source: Zookeys. 2021 Jun 24;1047:27–60. doi: 10.3897/zookeys.1047.61097 (PMC8249361; doi:10.3897/zookeys.1047.61097)
Supplement: Supplementary material 3 — Table S3. Variable loadings for principal components with Eigenvalues greater than 2, from morphometric characters corrected by SVL [file zookeys-1047-027-s003.docx]

**Table S3.** Variable loadings for principal components with Eigenvalues greater than 2, from morphometric characters corrected by SVL.

| Morphometric characters | Male | | Female | | |
| --- | --- | --- | --- | --- | --- |
|  | PC1 | PC2 | PC1 | PC2 | PC3 |
| SVL | -0.887 | -0.123 | -0.95 | 0.041 | 0.297 |
| HDL | 0.906 | -0.267 | -0.597 | 0.55 | 0.525 |
| HDW | 0.772 | 0.353 | 0.935 | -0.068 | 0.347 |
| SNT | 0.719 | -0.048 | 0.58 | -0.274 | 0.717 |
| ED | 0.383 | 0.568 | 0.261 | 0.765 | 0.256 |
| IOD | 0.569 | 0.149 | 0.708 | 0.564 | 0.391 |
| IND | 0.063 | 0.763 | 0.792 | 0.18 | 0.533 |
| TD | 0.177 | 0.399 | 0.306 | 0.794 | 0.52 |
| LAHL | 0.751 | 0.216 | 0.926 | 0.335 | -0.154 |
| LW | -0.603 | 0.562 | 0.957 | -0.153 | -0.17 |
| TL | 0.745 | 0.442 | 0.807 | 0.502 | -0.302 |
| THL | 0.85 | 0.042 | -0.164 | 0.947 | -0.201 |
| FL | 0.627 | 0.47 | 0.635 | 0.666 | -0.157 |
| TFL | 0.816 | 0.151 | 0.675 | 0.625 | -0.378 |
| HLL | 0.844 | 0.24 | 0.566 | -0.562 | 0.461 |
| TW | -0.579 | 0.608 | 0.967 | -0.227 | -0.089 |
| FIL | 0.838 | -0.349 | -0.486 | 0.638 | -0.369 |
| FIIL | 0.831 | -0.304 | -0.847 | 0.358 | 0.209 |
| FIIIL | 0.926 | -0.226 | -0.951 | 0.08 | 0.251 |
| FIVL | 0.893 | -0.255 | -0.846 | 0.504 | 0.163 |
| Eigenvalues | 9.641 | 3.391 | 4.274 | 6.265 | 2.85 |
| Percentage of total variance | 52.929 | 14.304 | 54.508 | 26.171 | 13.059 |
| Cumulative percentage | 52.929 | 67.230 | 54.508 | 80.679 | 93.738 |
